# Ge-Doped Boron Nitride Nanoclusters Functionalized with Amino Acids for Enhanced Binding of Bisphenols A and Z: A Density Functional Theory Study

**DOI:** 10.3390/ma17184439

**Published:** 2024-09-10

**Authors:** Chan-Fan Yu, Chia Ming Chang

**Affiliations:** Environmental Molecular and Electromagnetic Physics (EMEP) Laboratory, Department of Soil and Environmental Sciences, National Chung Hsing University, Taichung 40227, Taiwan; going30106@gmail.com

**Keywords:** boron nitride (B_12_N_12_) nanocluster, germanium, amino acid, bisphenol compound, estrogen-related receptor gamma, density functional theory

## Abstract

This study uses density functional theory to investigate boron nitride nanoclusters functionalized with amino acids for enhanced binding of bisphenols A (BPA) and Z (BPZ) to mimic the estrogen-related receptor gamma. Three categories of nanoclusters were examined: pristine B_12_N_12_, and those which were germanium-doped for boron or nitrogen. The study reveals that hydrogen bonding patterns and molecular stability are significantly influenced by the type of functional group and the specific amino acids involved. Ge-doping generally enhances the binding stability and spontaneity of the nanocluster–amino acid–bisphenol complexes, with Glu 275 emerging as the most stable binding site. The analysis of electronic properties such as energy gap, ionization potential, electron affinity, and chemical hardness before and after bisphenol binding indicates a general trend of increased reactivity, particularly in Ge-doped nanoclusters. The findings highlight the potential of these nanocluster composites in applications requiring high reactivity and electron mobility, such as pollutant removal and drug delivery.

## 1. Introduction

Previous studies have demonstrated that the adsorption of amphetamine (AMP) drugs onto nanoclusters results in significant alterations in their electronic properties, particularly a reduction in the bandgap, which can serve as an indicator of electrochemical signals [1]. DFT calculations indicate that the interaction between B_12_N_12_ nanoclusters and glycine amino acid leads to notable changes in their spectroscopic and electronic characteristics, with B_12_N_12_ exhibiting stronger interactions than B_16_N_16_ [2]. Further DFT studies reveal that the interaction of cysteine with single-walled BC_2_N nanotubes (BC_2_NNTs) is favorable in various media, with spontaneous dissolution in water particularly underscoring the system’s stability [3]. Subsequent research underscores the spontaneous dissolution of cysteine in water when interacting with BC_2_NNTs, further highlighting its application potential [4]. Moreover, the adsorption behavior of cysteine on pure B_12_N_12_ nanoclusters has been investigated using DFT and TD-DFT techniques, offering deep insights into the adsorption mechanisms of amino acids [5]. Previous research has identified that the complexation of donor-acceptor groups on B_12_N_12_ nanoclusters provides an energetic advantage and significantly reduces the HOMO-LUMO bandgap, emphasizing the material’s substantial potential in electronic device applications [6].

The recognition and detection of bioactive molecules, such as amino acids, on low-dimensional carbon-based materials depend on their interaction patterns and regulatory mechanisms, which are vital for biosensing applications [7]. A Ge-doped boron nitride nanotube (BNNT) sensor was developed to detect CO and NO, with findings showing a significant enhancement in the chemical adsorption of these gases on the doped BNNT [8]. In adsorption systems involving BNNT and amino acids, π-π and H-π stacking interactions were found to be the main stabilizing forces, with no bonding interactions observed [9]. Al_12_N_12_ and B_12_N_12_ nanocages have been identified as promising biomolecular sensor materials due to their high sensitivity to amino acids, which significantly alters their electronic properties during the adsorption process [10]. Research on the adsorption of various amino acids on carbon nanotubes (CNTs) indicates that the binding strength of zwitterionic glycine to CNTs is significantly higher than that of nonionic glycine, which is critical for improving detection sensitivity [11]. Density functional theory (DFT) calculations have shown that cysteine amino acid functionalized carbon nitride nanotubes (f-C_3_NNTs) perform exceptionally well as delivery systems for the anticancer drug thioguanine, highlighting their potential in medical applications [12]. Furthermore, the strong adsorption of tryptophan on BC_2_NNTs suggests its suitability as a delivery agent for tryptophan-based drugs, with particularly promising applications in biological environments [13].

Molecular dynamics simulations have revealed that lysine (Lys) molecules have a strong affinity for boron nitride nanosheet (BNNS) due to the presence of an alkyl tail, while aspartic acid (Asp) molecules exhibit repulsion because of their COO^-^ group; these findings are corroborated by free energy analysis [8]. Geometric optimization further suggests that the adsorption of proline on boron nitride cages is both exothermic and spontaneous, highlighting the stability of this structure in chemical reactions and its practical feasibility in experimental settings [14]. Structural optimization and electronic property analyses reveal that Fe atoms in amino acids were utilized to modify nanoscale zero-valent iron (AA@Fe^0^). This modified material was then applied in the Fenton-like degradation of organic solvents, specifically tributyl phosphate (TBP) (AA@Fe^0^-TBP); AA complexes tend to transfer charge with the -COO and -NH_3_ groups on the α-carbon of amino acids, which is crucial for improving sensing performance [15]. During adsorption, GABA and Glu molecules transfer charge to the BNNS structure, supporting the potential use of Fe- and Cu-doped BNNS as sensors for these molecules [16]. Research has also shown that complexes formed between Arg amino acids and graphene and BN nanosheets are highly stable and release energy during adsorption, making them valuable for applications in biosensors and drug delivery systems [17]. Through evaporation-induced self-assembly, four amino acids were integrated into cellulose nanocrystals to create chiral nematic films, which demonstrate significant potential in biosensing applications [18]. Additionally, chemical modification of pH-sensitive fluorescent nanoparticles using Fourier-transform infrared spectroscopy (FTIR), nuclear magnetic resonance (NMR), and X-ray photoelectron spectroscopy (XPS) has revealed their extensive potential for pH sensing applications [19].

The adsorption of glycine on B_12_N_12_ and B_16_N_16_ nanoclusters in water causes more significant changes in their electronic properties compared to interactions with other functional groups, suggesting that these clusters have potential for biomolecular sensing applications [2]. Moreover, amino acid functionalization not only improves the transport efficiency of biopharmaceuticals, but also enhances drug adsorption efficiency on C_4_B_32_ clusters, which is crucial for advancing nanodrug delivery systems [20]. Computational studies indicate that the zwitterionic form of serine exhibits higher binding stability on B_12_N_12_ fullerenes than its neutral form, emphasizing its potential use in designing biomolecular sensors [21]. The adsorption properties of tryptophan on BC_2_N nanotubes suggest that these nanotubes could serve as effective delivery agents for tryptophan-based drugs, highlighting their broad applicability in drug delivery systems [13]. Research on the charge distribution of nitrogen-doped graphene sheets shows that pyridine nitrogen and hydrogenated nitrogen atoms exhibit a stronger affinity for amino acids, offering valuable theoretical insights for biosensor design [22]. Additionally, the interaction between negatively polarized oxygen atoms on graphene oxide and the positively polarized regions of amino acid molecules demonstrates their potential in amino acid detection [23]. The strong adsorption of CO and NO on Ge-doped BNNTs underscores their potential as gas sensors, further extending their use in environmental monitoring applications [24].

Computational studies show that Ge-doped BPNT demonstrates increased electrical conductivity during chemical adsorption, despite its relatively low binding energy, indicating its potential for sensing applications [25]. Furthermore, P-doped carbon nanotubes (CNTs) and Ge-doped BNNT have been identified as effective catalysts for N_2_O reduction, offering a new technological approach for environmental applications [26]. The strong binding capability of doped C atoms in BNNS and BNNTs during NO_2_ adsorption highlights their excellent performance, making them valuable for the development of environmental sensing materials [27]. The adsorption of methanol on Ga- and Ge-doped graphene structures results in a reduction of the HOMO-LUMO bandgap, further underscoring the potential of these doped materials in gas sensing [28]. Additionally, the adsorption of VCM on the surface of nanotubes can produce significant signals in electronic circuits, with BNGaNT considered one of the most promising materials for designing gas sensors [29].

Functionalizing nanoclusters with amino acids involves attaching specific amino acids to enhance their properties for applications in biomedicine, catalysis, and materials science. This process utilizes the unique characteristics of nanoclusters like B_12_N_12_, Ge-B_11_N_12_, and Ge-B_12_N_11_, along with the functional groups of amino acids, such as carboxyl (-COOH) and amine (-NH_2_), which interact to introduce functionalities like hydrophilicity or specific binding capabilities based on the amino acid side chains. Typically, functionalization involves chemically bonding amino acids to nanoclusters through covalent, coordination, or non-covalent interactions. For instance, attaching glutamate (Glu), arginine (Arg), and asparagine (Asn) can modify nanocluster properties through electrostatic and hydrogen-bonding interactions. The objective of this study is to investigate the binding interactions and electronic property modifications that occur when boron nitride (B_12_N_12_) nanoclusters, both pristine and Ge-doped, are functionalized with specific amino acids and subsequently bonded with bisphenols A and Z (BPA and BPZ). By analyzing how these interactions influence the stability, reactivity, and electron transfer characteristics of the resulting nanocluster–amino acid–bisphenol complexes, the study aims to provide deeper insights into the molecular mechanisms underlying these interactions. This research seeks to determine the impact of such functionalizations on the nanoclusters’ chemical behavior, with potential implications for their use in applications such as drug delivery, environmental remediation, and nanotechnology-based sensors. It is important to clearly communicate the scope and limitations of our study. To this end, we wish to clarify that our research focuses on model systems that have not yet been synthesized. We acknowledge that the likelihood of their future synthesis is currently uncertain, and the primary aim of this work is to explore their theoretical properties and potential applications. This clarification is intended to set appropriate expectations, emphasizing that our study is a theoretical exploration designed to guide potential future experimental efforts, should these systems demonstrate sufficient scientific or practical interest.

## 2. Computational Details

This study utilized density functional theory (DFT) to investigate the molecular interactions and binding structures of boron nitride (B_12_N_12_) nanoclusters functionalized with amino acids, focusing on their interactions with bisphenol compounds (BPA and BPZ). The research encompassed three nanoclusters: pristine B_12_N_12_, Ge-doped B_11_N_12_, and Ge-doped B_12_N_11_. All DFT calculations, including energy calculations, geometry optimizations, and hydrogen bonding analyses, were performed using the DMol^3^ module within the Material Studio software suite [30,31].

The study explored the binding interactions of two bisphenols—bisphenol A (BPA) and bisphenol Z (BPZ)—with the estrogen receptor, using crystallographic data from the Protein Data Bank (PDB: 2E2R and 2ZKC). Molecular docking was conducted using the AutoDock Vina Extended SAMSON Extension, targeting the entire protein structure, with the grid centered to encompass the full size of the estrogen receptor. The top 200 docking poses were assessed, with the best candidates selected based on a standard scoring function [32].

To identify the optimal binding modes for the amino acids Glu 275, Arg 316, and Asn 346 within the crystal structures of 2E2R and 2ZKC, and to functionalize these groups (BPA and BPZ) onto B_12_N_12_, Ge-B_11_N_12_, and Ge-B_12_N_11_ nanoclusters, a series of computational steps were undertaken. First, a detailed analysis of the crystal structures was conducted to pinpoint the active sites of the selected amino acids and assess their binding potential with BPA or BPZ. This involved crystallographic data analysis and molecular docking simulations to determine the most stable binding configurations. Subsequently, docking tools were employed to model the binding of BPA or BPZ with 2E2R and 2ZKC, followed by optimization of the binding sites to identify the most stable amino acid–bisphenol interactions. The resulting amino acid–bisphenol complexes were then modeled and positioned on the surfaces of B_12_N_12_, Ge-B_11_N_12_, and Ge-B_12_N_11_ nanoclusters. Energy minimization and structural optimization were performed using DFT calculations to ensure that the functionalized nanocluster structures achieved their lowest energy states. This approach facilitated the selection of optimal binding modes for Glu 275, Arg 316, and Asn 346, and the subsequent bindings of BPA and BPZ onto the nanocluster composites.

While we aimed to explore a representative set of initial structures, the absence of structural similarity between the BN nanoclusters and the estrogen receptor might mean that some configurations were not fully explored. We will also mention that this is a limitation of the current study, and that future work could involve a more comprehensive exploration of the configurational space to ensure that all potential binding modes are considered.

The initial geometries of the nanocluster composites were optimized using the Generalized Gradient Approximation (GGA) with the Perdew–Burke–Ernzerhof (PBE) functional and a double numerical plus D-functions (DND) basis set [33]. For adsorption complexes, the basis set incorporated the relativistic effective core potential (ECP) with small-core pseudopotentials. Furthermore, the conductor-like screening model (COSMO) [34,35] was used to account for solvent effects under aqueous conditions, with the dielectric constant ε set to 78.54, corresponding to water. We employed the DFT-D approach using the Grimme (G06) scheme to correct the DFT energy for dispersion effects. Dispersion corrections were included from the outset in both the structural optimizations and the binding energy calculations [36]. The convergence criteria for both the electronic self-consistent field (SCF) and the geometric optimization were determined through a series of preliminary tests. We set the SCF convergence criterion to 1.0 × 10^−5^ Hartree to ensure a high degree of accuracy in the electronic structure calculations. For the geometric optimization, we adopted a force convergence criterion of 1.0 × 10^−3^ Hartree/Å. These criteria were selected based on a detailed analysis that aimed to achieve a balance between computational efficiency and the precision required for capturing the subtle interactions within the functionalized nanoclusters. The smearing value was chosen based on the need to ensure electronic convergence, particularly for systems with partially filled electronic states. We employed a thermal smearing method with a value of 0.005 Hartree. This value was selected after testing several smearing parameters to balance between achieving reliable electronic occupation distributions and minimizing the impact on the total energy. The chosen smearing value provided stable and consistent results across all calculations. The effects of Ge-doping on electronic properties and binding interactions were meticulously modeled.

Hydrogen bonding interactions were characterized by examining bond lengths and binding conformations, crucial for determining the stability and reactivity of the complexes. Stronger interactions, indicated by shorter hydrogen bonds, were associated with increased molecular stability. Binding energies (ΔE) were calculated to evaluate the stability of the nanocluster composite–bisphenol complexes. These energies were obtained by subtracting the sum of the energies of the isolated nanocluster composites (nanoclusters functionalized with amino acids) and bisphenol compounds from the total energy of the bisphenol-bonded nanocluster composites.

The study also analyzed electronic properties, including the energy gap (GAP), ionization potential (I), electron affinity (A), electronegativity (χ), and chemical hardness (η), both before and after nanocluster composites bonded with BPA and BPZ. These properties provided insights into the electronic behavior of the composites following bisphenol bindings. The relationships between energy changes (ΔE) and Hard and Soft Acids and Bases (HSABs) parameters were examined [37,38]. Correlation coefficients were calculated to evaluate the strength and nature of these relationships, offering valuable insights for the design and application of these nanoclusters across various fields.

## 3. Results and Discussion

### 3.1. Binding Structures between Boron Nitride Nanoclusters Functionalized with Amino Acids and Bisphenols

This study investigated the molecular structures of various nanoclusters, categorized into three groups: pristine boron nitride (B_12_N_12_) nanoclusters, Ge doped for B in B_12_N_12_, and Ge doped for N in B_12_N_12_. The binding interactions between these nanoclusters, functionalized with specific amino acids, and bisphenol compounds (BPA and BPZ) were analyzed to understand the impact of these bindings on molecular stability and hydrogen bonding.

**Figure 1:**
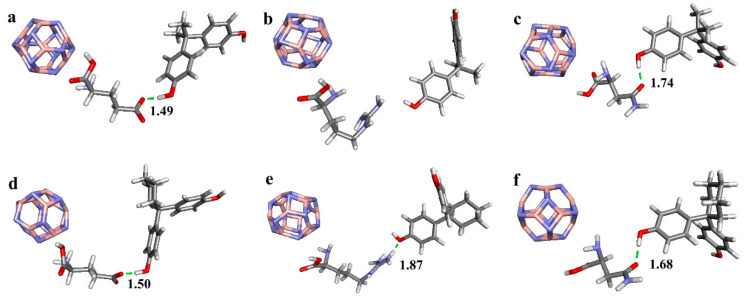
Optimized geometries of B_12_N_12_ nanocluster composites of (**a**) B_12_N_12__2E2R Glu 275_BPA, (**b**) B_12_N_12__2E2R Arg 316_BPA, (**c**) B_12_N_12__2E2R Asn 346_BPA, (**d**) B_12_N_12__2ZKC Glu 275_BPZ, (**e**) B_12_N_12__2ZKC Arg 316_BPZ and (**f**) B_12_N_12__2ZKC Asn 346_BPZ. (The numbers in the figure represent the distances between key atoms or groups within the molecular structures, measured in angstroms (Å)).

The optimized geometries are revealed in Figure 1. For BPA, it was observed that Arg 316 does not form hydrogen bonds in any of the nanocluster composites, potentially due to unfavorable binding conformations. However, Glu 275 consistently forms strong hydrogen bonds through its carboxyl group’s oxygen atom bonding with the hydrogen atom of the phenolic OH group in BPA. Asn 346 also participates in hydrogen bonding, using the oxygen atom of its amide group to interact with the phenolic OH group. Among all the examined cases, the bond between Glu 275 and BPA is the shortest (1.49 Å) in the pristine B_12_N_12_ composites, indicating a strong hydrogen bond and enhanced structural stability.

**Figure 2:**
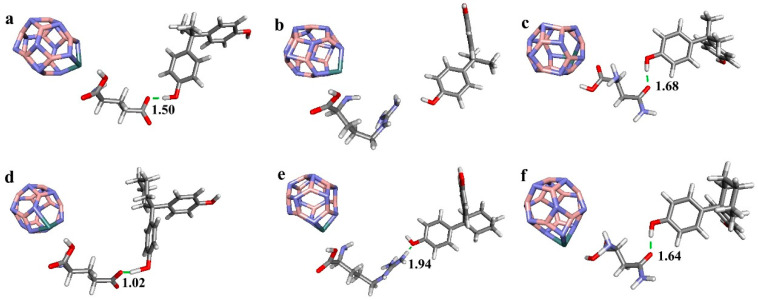
Optimized geometries of Ge-B_11_N_12_ nanocluster composites of (**a**) Ge-B_11_N_12__2E2R Glu 275_BPA, (**b**) Ge-B_11_N_12__2E2R Arg 316_BPA, (**c**) Ge-B_11_N_12__2E2R Asn 346_BPA, (**d**) Ge-B_11_N_12__2ZKC Glu 275_BPZ, (**e**) Ge-B_11_N_12__2ZKC Arg 316_BPZ and (**f**) Ge-B_11_N_12__2ZKC Asn 346_BPZ. (The numbers in the figure represent the distances between key atoms or groups within the molecular structures, measured in angstroms (Å)).

When Ge is doped for B in the B_12_N_12_ nanocluster composites (Figure 2), the hydrogen bonding pattern changes slightly. For BPA, Arg 316 still does not form hydrogen bonds, while Glu 275 and Asn 346 maintain their bonding patterns, similar to those in pristine nanoclusters. The bond between Glu 275 and BPA remains relatively short (1.50 Å), suggesting high stability. In contrast, the hydrogen bonding sites between amino acids and BPZ in Ge-doped nanocluster composites differ from the pristine composites, particularly in the interactions involving Arg 316, which forms hydrogen bonds through the hydrogen atom of its guanidino group.

**Figure 3:**
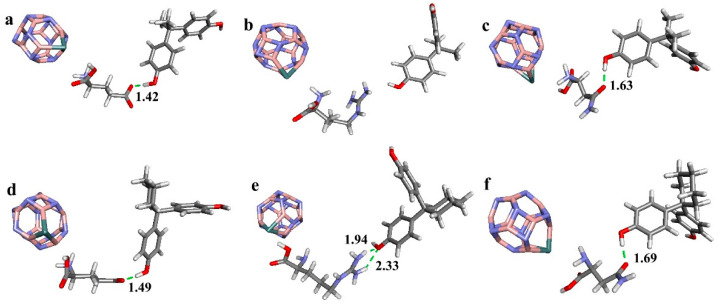
Optimized geometries of Ge-B_12_N_11_ nanocluster composites of (**a**) Ge-B_12_N_11__2E2R Glu 275_BPA, (**b**) Ge-B_12_N_11__2E2R Arg 316_BPA, (**c**) Ge-B_12_N_11__2E2R Asn 346_BPA, (**d**) Ge-B_12_N_11__2ZKC Glu 275_BPZ, (**e**) Ge-B_12_N_11__2ZKC Arg 316_BPZ and (**f**) Ge-B_12_N_11__2ZKC Asn 346_BPZ. (The numbers in the figure represent the distances between key atoms or groups within the molecular structures, measured in angstroms (Å)).

In Ge-doped-for-N nanocluster composites (Figure 3), the hydrogen bonding patterns exhibit further variations. For BPZ, Glu 275 continues to form hydrogen bonds through its carboxyl group’s oxygen atom, while Arg 316 forms two hydrogen bonds using its guanidino group’s hydrogen atoms. Interestingly, the shortest hydrogen bond is observed between Glu 275 and BPA (1.42 Å) in this configuration. However, Arg 316’s dual hydrogen bonds with BPZ suggest the need for additional energy calculations to determine the highest stability among these interactions.

The hydrogen bonding patterns between nanoclusters functionalized with amino acids and bisphenol compounds are significantly influenced by the functional groups of the amino acids and the nature of the nanocluster modifications. Notably, guanidino groups in Arg 316 can form multiple hydrogen bonds under specific conditions, while in some configurations Arg 316 does not participate in hydrogen bonding with BPA.

### 3.2. The Binding between Nanoclusters and Amino Acids under the Influence of Bisphenol Compounds

Nanoclusters play a dual role in drug delivery and monitoring environmental pollutants by contributing to the structural stability and balance of complexes. This study explored how nanoclusters, functionalized with amino acids, interact with bisphenol compounds, focusing on the stabilization mechanisms involved.

For Glu 275, the study found that all three types of nanoclusters—pristine B_12_N_12_, Ge-doped-for-B, and Ge-doped-for-N—stabilize this amino acid through interactions with a six-membered ring structure. In pristine B_12_N_12_ nanoclusters, stability is achieved through π-lone-pair interactions and hydrogen bonds with the nitrogen atoms of B_12_N_12_. However, an unfavorable bump interaction between the carboxyl group’s oxygen atom and the nitrogen atom slightly affects stability. In Ge-doped-for-B nanoclusters, stability is primarily maintained through interactions with Ge atoms, without specific bonding observed. In Ge-doped-for-N nanoclusters, the stabilization mechanism is similar, but is again affected by the same unfavorable bump interaction.

In BPA complexes, stability with Glu 275 is consistently achieved through interactions with the six-membered ring structure of the nanoclusters. In pristine B_12_N_12_, stability is mainly provided by interactions with the carboxyl and amino groups of Glu 275. Ge-doped-for-B nanoclusters introduce metal-acceptor interactions between the Ge atom and the nitrogen atom of the amino group, enhancing stability. Similar interactions are observed in Ge-doped-for-N nanoclusters.

For Arg 316, stability is simpler, and primarily involves interactions with the carboxyl and amino groups of the amino acid in pristine B_12_N_12_ nanoclusters. In BPA complexes, stability is further supported by π-donor interactions between the amino group’s hydrogen atoms and the carboxyl group with the nanocluster. In Ge-doped-for-B nanoclusters, stability involves weaker carboxyl interactions and metal-acceptor interactions between the amino group’s nitrogen atom and the Ge atom. In Ge-doped-for-N nanoclusters, stability is relatively weak for both BPA and BPZ complexes.

For Asn 346, stability in BPZ complexes with pristine B_12_N_12_ nanoclusters is weak, relying on interactions with the carboxyl and amino groups. However, in BPA complexes, stability is enhanced by hydrogen bonding between the carboxyl group’s hydrogen atom and the nanocluster’s six-membered ring structure. Ge-doped-for-B nanoclusters provide better stability in BPA complexes through two hydrogen bonds, while stability in BPZ complexes remains weak. Ge-doped-for-N nanoclusters exhibit weak interactions in both BPA and BPZ complexes.

Nanoclusters generally stabilize amino acids through interactions with their amino and carboxyl groups. The strength and type of these interactions vary, depending on the amino acid’s functional groups and the modifications in the nanocluster, offering insights into how these interactions can be leveraged for detecting bisphenols bound to nanocluster composites.

### 3.3. Energy Changes (ΔE) in Nanocluster–Amino Acid–Bisphenol Complexes

The binding energies presented in Table 1 reveal the energy changes (ΔE) associated with the functionalization of nanoclusters with amino acids (nanocluster composites), followed by the subsequent binding with bisphenol A (BPA) and bisphenol Z (BPZ). All binding energies are negative, indicating exothermic reactions, which suggest that the formation of these complexes is thermodynamically favorable. Notably, the Ge-doping into the nanocluster composites results in more negative binding energies, implying enhanced stability and spontaneity of the resulting complexes. The most stable binding interaction is observed with the Ge-B_11_N_12__2ZKC Glu 275 nanocluster composite bound to BPZ, with a ΔE of −45.37 kcal/mol.

Ge-B_11_N_12__2ZKC exhibited the most negative average ΔE (−41.59 kcal/mol), indicating that it consistently facilitates highly exergonic reactions, characterized by significant energy release, making it the most efficient nanocluster among those studied. Other Ge-doping nanoclusters, such as Ge-B_11_N_12__2E2R class (−35.55 kcal/mol) and Ge-B_12_N_11__2E2R class (−35.18 kcal/mol), also showed strongly negative ΔE values, although slightly less exergonic than the Ge-B_11_N_12__2ZKC class, suggesting that Ge-doping generally enhances the exergonic nature of the reactions. In contrast, the pristine nanoclusters, the B_12_N_12__2ZKC class (−16.37 kcal/mol) and the B_12_N_12__2E2R class (−15.95 kcal/mol), displayed the least negative ΔE values, indicating that these reactions are less exergonic, releasing less energy and reflecting lower efficiency in stabilizing the complexes compared to their Ge-doped counterparts.

Glu 275 consistently exhibited the largest negative ΔE across all molecule types, particularly with the Ge-B_11_N_12__2ZKC class (−45.37 kcal/mol) and the Ge-B_11_N_12__2E2R class (−39.80 kcal/mol), making it the most efficient residue for facilitating exergonic reactions and highlighting its critical role in stabilizing the complexes. Asn 346 also contributed significantly to energy release, with an average ΔE of −31.79 kcal/mol, demonstrating its importance, though it was less effective than Glu 275. In contrast, Arg 316 showed the least negative average ΔE (−25.95 kcal/mol) and the smallest variation in ΔE values, indicating more consistent but less-exergonic interactions, which contribute less to overall energy release compared to Glu 275 and Asn 346.

BPZ generally resulted in more exergonic interactions, with an average ΔE of −32.62 kcal/mol, compared to BPA, which had an average ΔE of −28.89 kcal/mol. The greater negativity of ΔE for BPZ indicates that it is a more effective adsorbate, likely due to stronger interactions and more favorable binding properties. This makes BPZ a preferable choice in applications where maximizing energy release is desired.

The study concludes that Ge-doping nanoclusters, particularly Ge-B_11_N_12_ and Ge-B_12_N_11_, have significant potential for promoting energy release, as evidenced by their dominant performance in exergonic reactions. The involvement of Glu 275 and Asn 346 residues is particularly crucial, as they consistently contribute to the high-energy interactions observed. These findings suggest that the strategic doping of Ge in nanoclusters can enhance their efficiency and stability, making them highly suitable for applications in environmental remediation and drug delivery. The results underscore the importance of residue-specific interactions and the choice of adsorbates in optimizing the performance of nanocluster-based systems.

### 3.4. Analysis of HSAB Parameters and Nanocluster–Amino Acid Interactions

The study of HSABs (Hard and Soft Acids and Bases) parameters provides critical insights into the reactivity and stability of boron nitride nanoclusters functionalized with amino acids (Table 2).

The interaction between Glu 275 and boron nitride nanoclusters results in the most significant reduction in bandgap (GAP), indicating a marked increase in the reactivity of these structures, which suggests that the electronic environment of Glu 275 is particularly sensitive to modifications in the nanocluster. The Ge-doping for B or N in the nanoclusters leads to a substantial decrease in GAP across all amino acids, indicative of enhanced reactivity with Ge-doping. Notably, when Ge dopes N modified with Glu 275, the reactivity hierarchy changes, with the 2ZKC class becoming more reactive compared to the 2E2R class after doping. The ionization potential (I), which measures the energy required to remove an electron, increases significantly with the modification of nanoclusters, particularly when modified with Asn 346, where it rises to 6.052 eV for the 2E2R class and 6.120 eV for the 2ZKC class after Ge-doping. This suggests increased stability and resistance to electron loss in these modified structures, potentially benefiting certain applications. Electron affinity (A), indicating the tendency of a structure to gain electrons, also increases following Ge-doping, implying that Ge-doped nanoclusters are more likely to accept electrons, thereby enhancing their reactivity. This suggests that Ge-doping not only stabilizes the nanocluster but also increases its ability to engage in electron transfer processes. The electronegativity (χ) of the nanoclusters, reflecting their ability to attract electrons, increases with the addition and modification of nanoclusters, particularly with Ge-doping. The highest electronegativity is observed in the Ge-doped nanoclusters interacting with 2ZKC Asn 346 (5.75 eV), highlighting the strong electron-attracting nature of this modified structure. Chemical hardness (η), positively correlated with GAP, is an indicator of a compound’s resistance to electron transfer and deformation. The modified nanoclusters exhibit lower hardness and GAP values, correlating with higher reactivity and a greater ability to transfer electrons, which aligns with the increased reactivity observed in these structures, especially following Ge-doping.

The results of this study clearly demonstrate that the doping of Ge into boron nitride nanoclusters significantly enhances their reactivity and electronic properties. The modifications lead to a decrease in GAP, an increase in ionization potential, and a rise in electron affinity, all of which contribute to the increased reactivity of the nanocluster–amino acid complexes. Structures involving Glu 275 exhibit the most pronounced changes, suggesting that this residue plays a critical role in determining the reactivity of the modified nanoclusters. Asn 346 also becomes more reactive with Ge-doping, particularly in the 2ZKC class, which shows the highest electronegativity.

### 3.5. Linear Correlation between Energy Changes (ΔE) and HSAB Parameters

**Figure 4:**
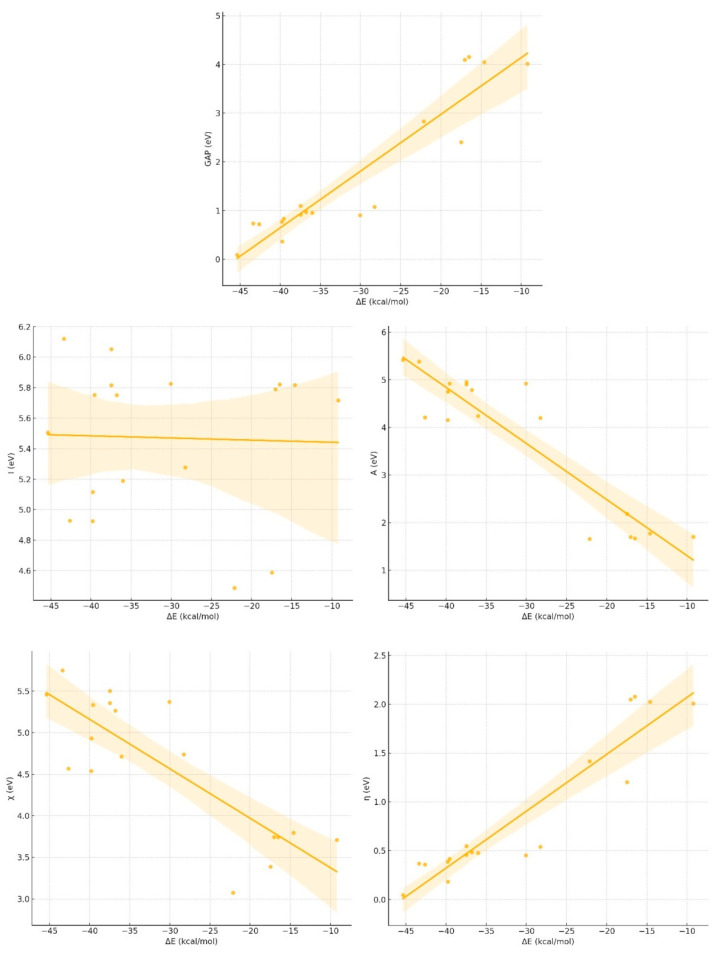
Linear correlation between the binding interaction energies of bisphenols with nanocluster composites (ΔE (kcal/mol)) and HSAB parameters (GAP (eV), the energy gap between the highest occupied molecular orbital (HOMO) and the lowest unoccupied molecular orbital (LUMO); I (eV), ionization energy required to remove an electron from a molecule; A (eV), electron affinity; χ (eV), electronegativity; η (eV), chemical hardness.

As the scatter plots show in Figure 4, a strong positive linear relationship was observed between the energy change (ΔE) and the energy gap (GAP), with a correlation coefficient of 0.935. This correlation suggests that as ΔE increases, the GAP also tends to increase, indicating that larger energy changes in reactions are generally associated with larger energy gaps, which often imply increased stability or reduced reactivity in nanocluster systems. Conversely, the correlation between ΔE and ionization energy (I) is very weak or negligible, as evidenced by a correlation coefficient of −0.033, indicating that ΔE does not significantly impact the ionization energy of the compounds studied. This implies that ionization energy remains relatively constant, despite variations in energy changes during reactions.

A strong negative linear relationship was observed between ΔE and electron affinity (A), with a correlation coefficient of −0.921. This suggests that as ΔE increases, electron affinity decreases, indicating that larger energy changes in a reaction are associated with a reduced tendency of the compounds to accept additional electrons. This decrease in electron affinity may reflect changes in the electronic structure, which lower the compounds’ ability to gain electrons, potentially affecting their reactivity and stability.

Similarly, a strong negative linear correlation exists between ΔE and electronegativity (χ), with a correlation coefficient of −0.825, indicating that higher ΔE values correspond to lower electronegativity. This suggests that higher energy changes in reactions are linked to reduced electronegativity, which may indicate a diminished ability of the compounds to attract electrons within bonds, potentially altering their chemical behavior.

A strong positive linear relationship was found between ΔE and chemical hardness (η), with a correlation coefficient of 0.935. This correlation suggests that as ΔE increases, the hardness of the compound also increases, indicating that larger energy changes are associated with compounds that are harder and more resistant to deformation or charge transfer. This enhanced hardness suggests that these compounds may be more stable and less reactive when it comes to electron distribution changes, which is crucial for maintaining structural integrity in various applications.

The study reveals that ΔE exhibits strong positive correlations with GAP and chemical hardness (η), suggesting that larger energy changes are associated with increased stability and resistance to electron transfer. These findings imply that nanocluster–amino acid complexes that undergo significant energy changes during reactions are likely to be more stable. Conversely, strong negative correlations are observed between ΔE and both electron affinity (A) and electronegativity (χ), indicating that larger energy changes correspond to reduced electron affinity and electronegativity. This reduction may reflect a decreased ability of the compounds to accept or attract electrons, which could influence their reactivity in specific chemical environments.

The lack of a significant correlation between ΔE and ionization energy (I) suggests that ionization energy remains relatively unaffected by the energy changes in the studied reactions. This finding highlights the potential for these nanocluster systems to maintain consistent ionization energy levels, despite variations in their electronic environments. These correlations provide valuable insights into the electronic properties of nanoclusters and their interactions with amino acids, guiding the design and selection of materials for applications that require specific stability and reactivity profiles.

### 3.6. Impact of Bisphenol A (BPA) and Bisphenol Z (BPZ) Binding on the Electronic Properties of Nanocluster Composites

The binding interaction of boron nitride nanocluster composites with bisphenol compounds, specifically Bisphenol A (BPA) and Bisphenol Z (BPZ), reveals significant alterations in their electronic properties, which directly affect the reactivity, stability, and electron transfer characteristics of these systems.

**Figure 5:**
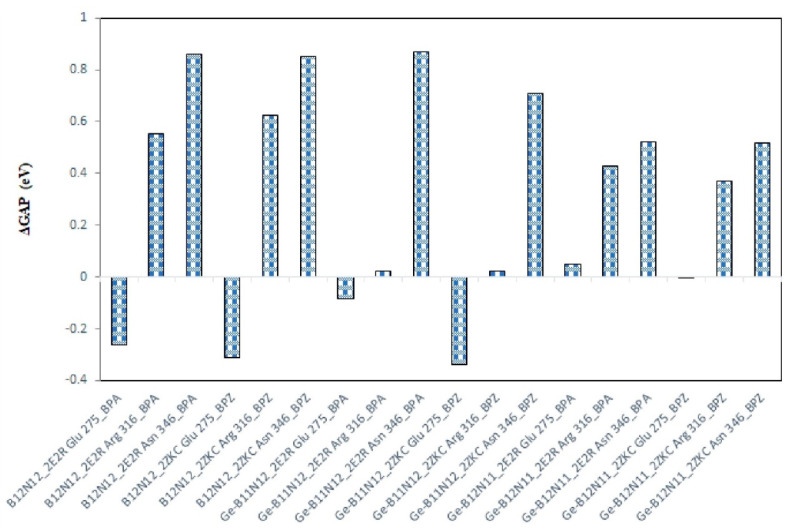
ΔGAP (eV) values after binding interaction of bisphenols with nanocluster composites.

The electronic properties of nanocluster composites show significant changes following bindings with BPA and BPZ, with several general trends observed (Table 3). There is a widespread reduction in the energy gap (GAP) across the majority of nanocluster structures, indicating increased reactivity. However, nanoclusters bound to Glu 275 deviate from this trend, maintaining stable or even increased GAP values, which suggests enhanced stability in these cases (Figure 5). Alongside the decrease in GAP, a general reduction in chemical hardness (η) is also observed, implying that these nanocluster composites are less resistant to deformation and more prone to electron transfer, which further correlates with their increased reactivity.

There is a slight decrease in both electronegativity (χ) and ionization energy (I) following the interactions, reflecting a diminished ability of the nanoclusters to attract bonding electrons and a reduced energy requirement for electron removal. These changes reinforce the overall trend towards heightened reactivity. Interestingly, electron affinity (A) remains relatively stable, suggesting that the bindings do not significantly impact the ability of the nanoclusters to accept electrons.

In a more detailed analysis, specific bindings result in pronounced reductions in GAP, such as the B_12_N_12__2E2R Asn 346 complex, where GAP decreases from 4.155 eV to 3.296 eV after interacting with BPA, indicating a shift towards higher reactivity. Similarly, Ge-B_12_N_11__2E2R Arg 316 shows a reduction in GAP from 0.906 eV to 0.476 eV, further underscoring this trend. The ionization energy (I) of most modified nanoclusters also decreases, suggesting that these structures require less energy to remove electrons and are more likely to engage in chemical reactions. Additionally, the reduction in electronegativity (χ), as seen in Ge-B_11_N_12__2E2R Asn 346, which drops from 5.504 eV to 5.203 eV after binding, aligns with the observed decreases in GAP and ionization energy. A significant decrease in hardness (η) is also noted, such as in the Ge-B_11_N_12__2ZKC Asn 346 complex, where hardness falls from 0.368 eV to 0.014 eV after interaction with BPZ, indicating that the bonded bisphenol compound becomes more reactive and less resistant to electron transfer or structural deformation.

Interestingly, structures bound to Glu 275 exhibit increased ionization potential following binding interaction with BPA and BPZ, indicating enhanced structural stability. These interactions make the Glu 275-bound composites more resistant to electron loss, potentially reducing their reactivity compared to other modified structures.

The interactions of nanocluster composites with BPA and BPZ generally lead to a decrease in GAP, ionization energy, electronegativity, and chemical hardness across most compounds, indicating a trend toward increased reactivity. These findings are crucial for understanding how specific chemical bindings, such as those involving BPA and BPZ, influence the electronic properties of nanocluster composites. This analysis highlights the importance of considering chemical bindings and their impact on the fundamental electronic properties of nanocluster composites, as these changes can significantly affect the functionality and suitability of nanocluster composites for specific technological applications.

## 4. Conclusions

This study provides a comprehensive examination of the binding interactions and electronic modifications in boron nitride (B_12_N_12_) nanoclusters functionalized with amino acids when interacting with bisphenol compounds (BPA and BPZ). The results demonstrate that the nature of hydrogen bonding between nanoclusters and amino acids is strongly influenced by the type of doping (Ge for B or N) and the specific amino acids involved. The hydrogen bond between Glu 275 and BPA was found to be the shortest across all composites, indicating strong interactions and greater stability. Conversely, Arg 316 exhibited limited or no hydrogen bonding with BPA, particularly in Ge-doped composites, underscoring the variability in bonding patterns based on structural modifications. The Ge-doping in nanoclusters generally enhanced the binding stability and spontaneity of the complexes, as reflected by more negative binding energies (ΔE). This enhancement suggests that Ge-doping nanoclusters are more effective in stabilizing amino acid–bisphenol complexes, particularly in interactions involving Glu 275. Additionally, the Ge-doping led to significant changes in electronic properties, including a decreased energy gap (GAP), increased ionization potential (I), and increased electron affinity (A), indicating an overall increase in reactivity, especially in complexes involving Glu 275 and Asn 346. Bindings with BPA and BPZ generally resulted in increased reactivity across most structures. Strong correlations were identified between energy changes (ΔE) and key HSAB parameters (GAP, A, χ, η), indicating that larger energy changes are associated with increased stability and resistance to electron transfer, while also correlating with reduced electron affinity and electronegativity.

## Figures and Tables

**Table 1:** The binding interaction energies of bisphenols with nanocluster composites.

	ΔE (kcal/mol)
B_12_N_12__2E2R Glu 275 + BPA→ B_12_N_12__2E2R Glu 275_BPA	−22.13
B_12_N_12__2E2R Arg 316 + BPA → B_12_N_12__2E2R Arg 316_BPA	−9.22
B_12_N_12__2E2R Asn 346 + BPA → B_12_N_12__2E2R Asn 346_BPA	−16.51
B_12_N_12__2ZKC Glu 275 + BPZ → B_12_N_12__2ZKC Glu 275_BPZ	−17.47
B_12_N_12__2ZKC Arg 316 + BPZ → B_12_N_12__2ZKC Arg 316_BPZ	−14.62
B_12_N_12__2ZKC Asn 346 + BPZ → B_12_N_12__2ZKC Asn 346_BPZ	−17.03
Ge-B_11_N_12__2E2R Glu 275 + BPA → Ge-B_11_N_12__2E2R Glu 275_BPA	−39.80
Ge-B_11_N_12__2E2R Arg 316 + BPA → Ge-B_11_N_12__2E2R Arg 316_BPA	−28.27
Ge-B_11_N_12__2E2R Asn 346 + BPA → Ge-B_11_N_12__2E2R Asn 346_BPA	−37.47
Ge-B_11_N_12__2ZKC Glu 275 + BPZ → Ge-B_11_N_12__2ZKC Glu 275_BPZ	−45.37
Ge-B_11_N_12__2ZKC Arg 316 + BPZ → Ge-B_11_N_12__2ZKC Arg 316_BPZ	−36.03
Ge-B_11_N_12__2ZKC Asn 346 + BPZ → Ge-B_11_N_12__2ZKC Asn 346_BPZ	−43.37
Ge-B_12_N_11__2E2R Glu 275 + BPA → Ge-B_12_N_11__2E2R Glu 275_BPA	−39.78
Ge-B_12_N_11__2E2R Arg 316 + BPA → Ge-B_12_N_11__2E2R Arg 316_BPA	−30.08
Ge-B_12_N_11__2E2R Asn 346 + BPA → Ge-B_12_N_11__2E2R Asn 346_BPA	−36.79
Ge-B_12_N_11__2ZKC Glu 275 + BPZ → Ge-B_12_N_11__2ZKC Glu 275_BPZ	−42.63
Ge-B_12_N_11__2ZKC Arg 316 + BPZ → Ge-B_12_N_11__2ZKC Arg 316_BPZ	−37.49
Ge-B_12_N_11__2ZKC Asn 346 + BPZ → Ge-B_12_N_11__2ZKC Asn 346_BPZ	−39.56

**Table 2:** The HSAB parameters before binding interaction of bisphenols with nanocluster composites ^a^.

	GAP (eV)	I (eV)	A (eV)	χ (eV)	η (eV)
2E2R	4.016	5.092	1.076	3.084	2.008
2ZKC	3.931	5.061	1.130	3.096	1.966
B_12_N_12__2E2R Glu 275	2.830	4.488	1.658	3.073	1.415
B_12_N_12__2E2R Arg 316	4.014	5.716	1.702	3.709	2.007
B_12_N_12__2E2R Asn 346	4.155	5.821	1.666	3.744	2.078
B_12_N_12__2ZKC Glu 275	2.404	4.588	2.184	3.386	1.202
B_12_N_12__2ZKC Arg 316	4.047	5.818	1.771	3.795	2.024
B_12_N_12__2ZKC Asn 346	4.097	5.790	1.693	3.742	2.049
Ge-B_11_N_12__2E2R Glu 275	0.771	4.925	4.154	4.540	0.386
Ge-B_11_N_12__2E2R Arg 316	1.077	5.277	4.200	4.739	0.539
Ge-B_11_N_12__2E2R Asn 346	1.096	6.052	4.956	5.504	0.548
Ge-B_11_N_12__2ZKC Glu 275	0.090	5.506	5.416	5.461	0.045
Ge-B_11_N_12__2ZKC Arg 316	0.952	5.189	4.237	4.713	0.476
Ge-B_11_N_12__2ZKC Asn 346	0.736	6.120	5.384	5.752	0.368
Ge-B_12_N_11__2E2R Glu 275	0.366	5.115	4.749	4.932	0.183
Ge-B_12_N_11__2E2R Arg 316	0.906	5.825	4.919	5.372	0.453
Ge-B_12_N_11__2E2R Asn 346	0.966	5.750	4.784	5.267	0.483
Ge-B_12_N_11__2ZKC Glu 275	0.718	4.928	4.210	4.569	0.359
Ge-B_12_N_11__2ZKC Arg 316	0.915	5.816	4.901	5.359	0.458
Ge-B_12_N_11__2ZKC Asn 346	0.833	5.752	4.919	5.336	0.417

^a^ The variables include GAP (eV), the energy gap between the highest occupied molecular orbital (HOMO) and the lowest unoccupied molecular orbital (LUMO); I (eV), ionization energy required to remove an electron from a molecule; A (eV), electron affinity, indicating the energy change when an electron is added; χ (eV), electronegativity, representing the ability to attract electrons; and η (eV), chemical hardness, measuring resistance to electron distribution changes.

**Table 3:** The HSAB parameters after binding interaction of bisphenols with nanocluster composites.

	GAP (eV)	I (eV)	A (eV)	χ (eV)	η (eV)
B_12_N_12__2E2R Glu 275_BPA	3.091	4.744	1.653	3.199	1.546
B_12_N_12__2E2R Arg 316_BPA	3.460	5.128	1.668	3.398	1.730
B_12_N_12__2E2R Asn 346_BPA	3.296	4.957	1.661	3.309	1.648
B_12_N_12__2ZKC Glu 275_BPZ	2.713	4.920	2.207	3.564	1.357
B_12_N_12__2ZKC Arg 316_BPZ	3.423	5.199	1.776	3.488	1.712
B_12_N_12__2ZKC Asn 346_BPZ	3.245	4.935	1.690	3.313	1.623
Ge-B_11_N_12__2E2R Glu 275_BPA	0.853	4.962	4.109	4.536	0.427
Ge-B_11_N_12__2E2R Arg 316_BPA	1.056	5.234	4.178	4.706	0.528
Ge-B_11_N_12__2E2R Asn 346_BPA	0.228	5.317	5.089	5.203	0.114
Ge-B_11_N_12__2ZKC Glu 275_BPZ	0.426	5.277	4.851	5.064	0.213
Ge-B_11_N_12__2ZKC Arg 316_BPZ	0.930	5.189	4.259	4.724	0.465
Ge-B_11_N_12__2ZKC Asn 346_BPZ	0.027	5.367	5.340	5.354	0.014
Ge-B_12_N_11__2E2R Glu 275_BPA	0.316	5.059	4.743	4.901	0.158
Ge-B_12_N_11__2E2R Arg 316_BPA	0.476	5.411	4.935	5.173	0.238
Ge-B_12_N_11__2E2R Asn 346_BPA	0.442	5.249	4.807	5.028	0.221
Ge-B_12_N_11__2ZKC Glu 275_BPZ	0.724	4.936	4.212	4.574	0.362
Ge-B_12_N_11__2ZKC Arg 316_BPZ	0.543	5.448	4.905	5.177	0.272
Ge-B_12_N_11__2ZKC Asn 346_BPZ	0.316	5.239	4.923	5.081	0.158

## Data Availability

The original contributions presented in the study are included in the article, further inquiries can be directed to the corresponding author.
